# COVID-19 Diagnostics, Tools, and Prevention

**DOI:** 10.3390/diagnostics10060409

**Published:** 2020-06-16

**Authors:** Mayar Allam, Shuangyi Cai, Shambavi Ganesh, Mythreye Venkatesan, Saurabh Doodhwala, Zexing Song, Thomas Hu, Aditi Kumar, Jeremy Heit, COVID-19 Study Group, Ahmet F. Coskun

**Affiliations:** 1 Wallace H. Coulter Department of Biomedical Engineering, Georgia Institute of Technology and Emory University, Atlanta, GA 30332, USA; mallam6@gatech.edu (M.A.); shuangyi@gatech.edu (S.C.); shambavi.ganesh@gatech.edu (S.G.); mvenkatesan7@gatech.edu (M.V.); spd@gatech.edu (S.D.); zsong91@gatech.edu (Z.S.); thomashu@gatech.edu (T.H.); akumar16@gatech.edu (A.K.); jheit3@gatech.edu (J.H.); kasfiakazi@gatech.edu (C.S.G.); 2 School of Electrical and Computer Engineering, Georgia Institute of Technology, Atlanta, GA 30332, USA; 3 H. Milton Stewart School of Industrial and Systems Engineering, Georgia Institute of Technology, Atlanta, GA 30318, USA; 4 Woodruff School of Mechanical Engineering, Georgia Institute of Technology, Atlanta, GA 30313, USA; 5 School of Chemical and Biomolecular Engineering, Georgia Institute of Technology, Atlanta, GA 30332, USA

**Keywords:** COVID-19, SARS-CoV-2, rapid testing, immunity, vaccines, 3D printing, do-it-yourself, digital tracking, machine learning, pandemic policy

## Abstract

The Coronavirus Disease 2019 (COVID-19), caused by the severe acute respiratory syndrome coronavirus-2 (SARS-CoV-2), outbreak from Wuhan City, Hubei province, China in 2019 has become an ongoing global health emergency. The emerging virus, SARS-CoV-2, causes coughing, fever, muscle ache, and shortness of breath or dyspnea in symptomatic patients. The pathogenic particles that are generated by coughing and sneezing remain suspended in the air or attach to a surface to facilitate transmission in an aerosol form. This review focuses on the recent trends in pandemic biology, diagnostics methods, prevention tools, and policies for COVID-19 management. To meet the growing demand for medical supplies during the COVID-19 era, a variety of personal protective equipment (PPE) and ventilators have been developed using do-it-yourself (DIY) manufacturing. COVID-19 diagnosis and the prediction of virus transmission are analyzed by machine learning algorithms, simulations, and digital monitoring. Until the discovery of a clinically approved vaccine for COVID-19, pandemics remain a public concern. Therefore, technological developments, biomedical research, and policy development are needed to decipher the coronavirus mechanism and epidemiological characteristics, prevent transmission, and develop therapeutic drugs.

## 1. Introduction

The Coronavirus Disease 2019 (COVID-19), caused by the novel coronavirus strain, known as the severe acute respiratory syndrome coronavirus-2 (SARS-CoV-2), emerged in 2019 and is considered to be a pandemic in the form of viral pneumonia [1]. In the last two decades, there have been several epidemics, including SARS-CoV in 2002–2003, H1N1 influenza in 2009, and MERS in 2012. The coronavirus family (CoV) are single-stranded RNA viruses that are found in different animals and can cross species barriers and use other mammals as hosts. These viruses have the potential to become a pandemic, as the World Health Organization (WHO) declares COVID-19 to be outbreaks of infectious disease crossing international boundaries, resulting in morbidity and mortality at a large scale [2]. These pandemics can affect the economy and cause social and political disruptions. This rise in pandemics can be attributed to global travel and the exploitation of the environment. For these outbreaks to subside and be prevented, there is an urgent need to identify emerging outbreaks and create policies to act accordingly [3]. Well-planned public health structures, such as the Centers for Disease Control and Prevention (CDC) and societal policies are needed to disseminate public health preparedness and guide the emergent response, as well as identify gaps in knowledge and solve them.

This paper presents diagnostic and prevention tools to control the novel COVID-19 pandemic. Herein, we discuss the biological basis of the disease progression and transmission routes, along with the historical background of similar viral infections. Similarities and differences between the novel SARS-CoV and other viruses from the coronavirus family are analyzed. Distinct methods by which the virus gets transmitted between carriers are discussed. Strategies to eliminate the current pandemic are analyzed by herd immunity, shield immunity, vaccine development, and proactive screening. An overview of the preventive tools and measures that can decrease the likelihood of the virus transmission are presented to highlight the proper use of personal protective equipment (PPE), including the masks, face shields, gloves, and ventilators. Three-dimensional printing for designing and manufacturing do-it-yourself (DIY) protective tools are discussed to rapidly democratize and deliver health-related equipment and supplies at a low cost. Interdisciplinary approaches are discussed to leverage machine learning, digital monitoring, and statistical simulations for modeling and predicting the spatial–temporal dynamics of COVID-19. Policies that regulate the process of reporting COVID-19 patients, developing vaccines, and categorizing the population based on immunity are investigated. The presented biological, technical, analytical, and regulatory framework will shed light on the strategies to diagnose, prevent, and regulate the impacts of COVID-19.

### 1.1. Coronaviruses

On 23 February 2020, the lockdown of Wuhan in China finally caught people’s attention and alarmed the public all over the world. The novel virus, Coronaviruses, is one of the members of the family Coronaviridae and has been identified in both avian hosts and various mammals. Since coronaviruses were considered as pathogens that only cause mild disease, people previously failed to pay enough attention until the outbreak of the severe acute respiratory syndrome (SARS) coronavirus (SARS-CoV) [4].

SARS-CoV is one of 36 coronaviruses in the family Coronaviridae within the order Nidovirales. The demand for consumption of animal proteins allowed for the transmission from animals to humans [5]. The animal-to-human and human-to-human transmission, as well as the lack of awareness in controlling infection, resulted in a large-scale spread over 8000 people, with a fatality rate of 10% in southern China in 2003. Horseshoe bats are the natural reservoir for SARS-CoV-like viruses, and civets can amplify the host and facilitate the transmission.

Another member of the family Coronaviridae is the Middle East respiratory syndrome coronavirus (MERS-CoV), which was first reported by Saudi Arabia in 2012 [6]. MERS coronaviruses can cause both human and veterinary outbreaks due to the ability of coronaviruses to recombine, mutate, and transmit between animals. Symptomatic MERS-CoV has an incubation period of 2 to 14 days, and symptoms include respiratory and enteric disease. Training and milking of camels have been associated with MERS-CoV infection, and people that consume raw camel milk or improperly cooked meat have a high risk of contracting MERS-CoV. The camel birthing season may result in the seasonal pattern of cases, which has a peak each year between April and June.

**Figure 1:**
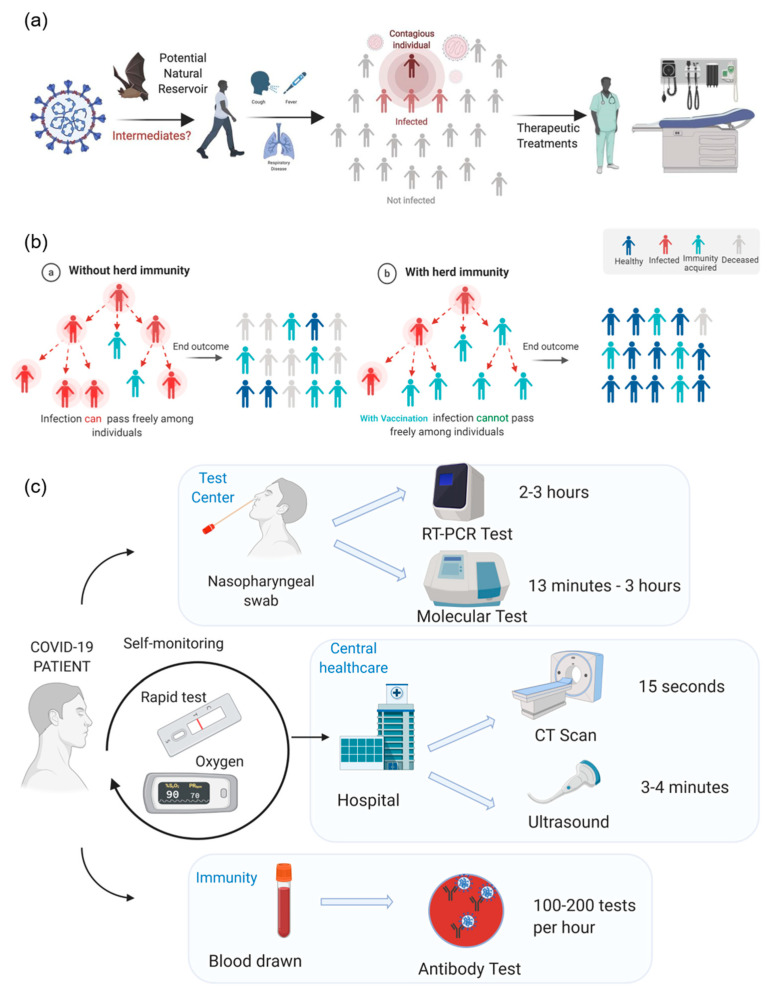
COVID-19 origins, transmission, immunity, and diagnostics. (**a**) Animals such as bats are a potential natural reservoir, and other intermediates facilitate the infection of coronavirus to humans. Human-to-human transmission is a common characteristic in three types of coronaviruses, severe acute respiratory syndrome coronavirus (SARS-CoV), SARS-CoV-2, and Middle East respiratory syndrome coronavirus (MERS-CoV). Even though the fatality rate is not lower than SARS-CoV and MERS-CoV, vaccines and treatments are needed; (**b**) Herd immunity plays an essential role in controlling the transmission of the disease. Without herd immunity, the virus will be available to get transferred from infected patients to any susceptible individual, resulting in a high infection rate and high mortality. On the other hand, herd immunity interrupts this chain of infection, resulting in better outcomes with less infected individuals and a significantly reduced the mortality rate; (**c**) The three main categories of COVID-19 diagnostics and the time-frame of each: laboratory test centers for current infection, medical screenings, antibody tests to determine immunity. Test centers use samples from nasopharyngeal swabs and test for COVID-19 infection via molecular or reverse transcription-polymerase chain reaction (RT-PCR) tests, with RT-PCR tests taking longer. Healthcare clinics and hospitals offer quick medical screenings via computed tomography (CT) scans or ultrasounds; ultrasounds are the safer option due to no radiation. The next step in rapid testing will be to test COVID-19 patients for antibodies against SARS-CoV-2 to see if the patient has the potential to fight reinfection and determine if the individual was previously sick.

All three types of the coronavirus have shown to be a severe acute respiratory syndrome; however, SARS-CoV-2 has a lower fatality rate of 2.3%, while SARS-CoV and MERS-CoV have 10% and 36%, respectively. The key host for SARS-CoV-2 is bats; however, the intermediate zoonotic sources need to be identified. The most common signs and symptoms for people with COVID-19 are fever and cough. Fever was observed in around 90% of infected patients. A typical characteristic of COVID-19 is pneumonia and people have displayed heterogeneous symptoms, such as shortness of breath or dyspnea, as well as muscle ache. SARS-CoV-2 can be transmitted human-to-human and is mainly through indirect or direct contact with mucous membranes in the mouth, eyes, or nose (Figure 1a). The droplets from coughing can penetrate the human body via nose or mouth. SARS-CoV-2 has a higher transmission rate than SARS-CoV, probably due to the genetic recombinant event in COVID-19. SARS-CoV-2 also has an incubation period from 3 to 7 days, but it could be as long as 24 days in rare cases [4].

### 1.2. Immunity

The chain of coronavirus infection happens when one person infects another and the cycle keeps going. This chain breaks when a significant proportion of the population has immunity against the viral or bacterial disease, through what is known as herd immunity [7,8]. This technique remains ineffective for all diseases such that the human body develops immunity against certain disorders after the first infection, or sometimes through vaccination. The same pattern applies to the newly emerging viral infection, COVID-19, due to the spread of the SARS-CoV-2 virus (Figure 1b). However, the biggest concern for COVID-19 is the balance between the natural rate of disease transmission, and social distancing principles such that the population acquires herd immunity after it gets significantly exposed to the virus that facilitates the elimination of the pandemic. However, with the strict social distancing guidelines, the population remains unexposed to the virus to develop herd immunity. Lastly, a recently developed framework, termed as shield immunity, builds a pandemic recovery based on central interactions of recovered individuals, exhibiting no virus, but with their antibodies detected in serological tests [9]. The shield immunity concept has the potential to enrich safer social networks through substitution of interactions to control the evolving dynamics of pandemics.

### 1.3. Vaccines

Drug Repurposing Against COVID-19: Recent drug discovery efforts have repurposed drugs that were previously used for other health conditions to treat COVID-19, and in particular, the combination of anti-malaria drugs, including Food and Drug Administration (FDA)-approved drugs, such as hydroxychloroquine and chloroquine [10,11]. However, these drugs may not be suitable for COVID-19 treatment because of their narrow therapeutic window, such that the difference between the effective dose and the toxic dose is significantly small. Given that there is not enough strong clinical data to support the alleged benefits from these drugs, it can worsen symptoms associated with COVID-19. There have been prior attempts to use antimalarial drugs in controlling other viral infections, including human immunodeficiency virus (HIV) and hepatitis C. For example, the antimalarial drug, ferroquine was proven to be a strong inhibitor of the hepatitis C virus, while another antimalarial drug, chloroquine showed weak efficacy against HIV infection [12,13,14]. Although antimalarial drugs can be effective against a number of viral infections, their response varies for all viruses and conditions. Thereby, more clinical trials are needed to judge the ability of antimalarial drugs in treating COVID-19 patients to further understand the potential benefits and risks of these drugs. For example, the Washington University School of Medicine in St. Louis recently launched a clinical trial to test the efficacy of the antimalarial drugs chloroquine and hydroxychloroquine alone and in combination with another drug, the antibiotic azithromycin [15]. These drugs are currently being tested on COVID-19 hospitalized patients at the Barnes-Jewish Hospital.

In addition to hydroxychloroquine and chloroquine, Remdesivir (GS-5734) is another drug that is being evaluated for the treatment of COVID-19. Remdesivir, as an inhibitor of the viral RNA polymerases, has broad-spectrum activity against SARS-CoV and MERS-CoV and has been identified as a potential candidate for the treatment of COVID-19. A double-blind, randomized, placebo-controlled trial was performed on patients that were hospitalized with COVID-19 and showed the benefits of Remdesivir in improving time to recovery and lowering respiratory tract infection. However, the trial was implemented in restricted locations and during a time of restricted travel. Since safety procedures limit the resources, research staff often monitored the visits remotely [16]. Although the compassionate treatment program in another preliminary report for evaluating the efficacy of Remdesivir demonstrated 68% of clinical improvement, the results were not comparable to that of the other reports due to the lack of randomized trials. The interpretation of the results is limited by the lack of viral load data collection, a non-uniform duration of therapy, and the small size of the cohort [17]. Therefore, these preliminary reports can only provide clinicians the option of Remdesivir, and further assessments on the safety and efficacy of Remdesivir and potential combinations with other therapeutic agents are required via randomized, placebo-controlled trials.

Favipiravir (Avigan) is another broad-spectrum antiviral drug that works by inhibiting the RNA polymerase machinery of RNA viruses, resulting in a nonviable virus phenotype. It was developed by Toyama Chemical in 2014 through screening chemical libraries for anti-viral activity against the influenza virus, and was approved and stockpiled in Japan against novel influence pandemics [18,19,20]. This drug was recently shown to be effective against other viral diseases, including Ebola and the novel coronavirus (SARS-CoV-2) [21]. Clinical trials in China have led the effort in testing the efficacy of Favipiravir in treating COVID-19 patients in February 2020. The study showed that Favipiravir resulted in a significant decrease in the median time to viral clearance and faster improvement in patients’ chest computed tomography (CT) scans [22]. However, this study was small and non-randomized, meaning that it needs to be supported by bigger, open-label, randomized, and controlled clinical trials to test the safety and efficacy of Favipiravir. Even though the drug was not initially approved in the USA for the treatment of influenza, several clinical trials have been designed to test its efficacy in treatment of COVID-19 patients across the entire country [23,24].

Similarly, Ivermectin is an anthelmintic drug that acts on parasites and roundworm infections. This drug was shown to be a promising candidate for treating viral diseases, including chikungunya and yellow fever [25]. Several clinical trials are underway after Ivermectin was proven to have in vitro activity against SARS-CoV-2. Ivermectin led to significant reduction in viral RNA shortly after treatment [26]. However, the concentrations needed to achieve these promising in vitro results were much higher than those recommended and approved for the treatment of parasitic infections. Thereby, the FDA issued a warning against the inappropriate use of Ivermectin for the treatment of COVID-19 patients [27].

Vaccine Development: Another method to eliminate this pandemic is through vaccines that can solve the problem from its roots by protecting healthy individuals from catching and transferring infections. Since the genomic data of the novel SARS-CoV-2 became available in January 2020, there has been an increasing global interest in vaccine development. As of April 2020, there have been 115 vaccine candidates against SARS-CoV-2 worldwide [28]. A subset of these vaccines is at the clinical phase and are currently being tested on human subjects. These vaccine candidates include mRNA-1273 from ModernaTX [29]. mRNA-1273 utilizes an emerging technology in vaccine development and delivery of a lipid nanoparticle (LNP) mRNA vaccine that encodes for the S protein on the novel SAR-CoV-2 virus. Another successful vaccine candidate is Ad5-nCoV from CanSino that is an adenovirus vector encoding for the S protein [30]. Furthermore, INO-4800 was developed by Inovio Pharmaceuticals, which is a DNA plasmid that encodes for the S protein of the virus [31]. Finally, the Shenzhen Geno-Immune Medical Institute developed two vaccine candidates, LV-SMENP-DC and pathogen-specific APC, which are lentiviral vectors designed to express proteins of interest to deactivate the virus [32].

One of the contributing factors to virus infectivity and virulence is a phenomenon called antibody-dependent enhancement (ADE). ADE refers to the viruses binding to specific antibodies of the surface of host cells that enhance their entry and replication. This phenomenon was observed in many viruses, including the yellow fever virus, zika virus, and coronaviruses [33]. ADE can adversely impact the process of vaccine development because vaccines could trigger the production of antibodies that viruses could use to gain access to host cells, so the vaccine could worsen the condition that it was designed to fight. Thus, ADE is a major potential challenge for COVID-19 vaccine development, as ADE was observed in other types of coronaviruses that resulted in previous pandemics, including SARS-CoV and MERS-CoV. For example, there were several vaccine candidates for the SARS-CoV pandemic, including whole inactivated viruses, DNA, subunits, and vectored vaccines. Although these vaccines were able to halt the replication of the SARS-CoV virus in infected cells, they granted viral access to macrophages through ADE, which eventually caused serious lung infections [34,35]. The SARS vaccine candidates resulted in enhanced hepatitis, increased morbidity, and stronger inflammatory responses that were attributed to ADE [36]. Similarly, the MERS-CoV virus was shown to utilize the cellular receptors that mimic viral receptors to gain access to host cells, resulting in enhanced infection [37]. Little is known about whether ADE will present a challenge to target the SARS-CoV-2 due to gained access to immune cells or due to prior coronavirus infections, and it is currently an active area of investigation [38].

## 2. COVID-19 Testing, Prevention Tools, and Tracking

### 2.1. Rapid Testing

Test results for COVID-19 can lead to the initial steps in identifying the scale of the pandemic. With the data from the test results, mathematical models can be created to aid governors’ decisions about the length of time for stay-at-home orders. Previously, reverse transcription-polymerase chain reaction (RT-PCR) tests were the diagnostic method of choice. However, these methods are not ideal for rapid diagnosis due to their long wait times of two to three hours, as well as the need for expensive laboratory equipment and trained professionals (Figure 1c).

For the rapid diagnosis of current infection, COVID-19 testing stations use molecular tests to determine the presence of SARS-CoV-2. These tests rely on the genetic sequence of SARS-CoV-2 and use PCR (Figure 1c). Under the Emergency Use Authorization (EUA), many labs can utilize their existing machines for detection, which vary in terms of multiplexing, complexity, throughput, and turnaround times. For example, Genmark’s ePlex Respiratory Pathogen instrument can detect common pathogens in the nasopharyngeal swab specimens. Cobas^®^ SARS-CoV-2 on the cobas^®^ 6800 [39] (Roche Molecular Diagnostics, Pleasanton, CA, USA) and Xpert^®^ Xpress SARS-CoV-2 [40] (Cepheid, Sunnyvale, CA, USA) give high-volume and automated throughput using the Lightmix^®^ Kit. While these devices can use specimens other than nasopharyngeal swabs, the Cobas^®^ 6800 takes 3.5 h and the Xpert^®^ Xpress SARS-CoV-2 takes 45 min. Currently, Abbott’s ID Now, a rapid molecular in vitro diagnostic test utilizing an isothermal nucleic acid amplification technology, has a COVID-19 feature for testing, which takes less than 13 min [41]. A nasopharyngeal, throat, and nasal swab can be used. Although there is considered to be a sensitivity of 90% with these molecular tests, there still stands the risk and repercussions of false-negative tests with these current devices.

With COVID-19 being considered a respiratory infection, several studies focus on using medical screening lung scans to track the progress of the disease in central healthcare infrastructures. One study in Wuhan, China focused on the progression of COVID-19 in the lungs of 81 patients, and used CT scans to track the progress [42]. All patients with and without symptoms of the “viral pneumonia” showed abnormal chest CTs, specifically as bilateral, subpleural, ground-glass opacities with air bronchograms, ill-defined margins, and a slight predominance in the lower right lobe. As the patient heals, the patient’s lungs show improvements in the lung lesions. This medical screening was considered to be quicker and more sensitive than the RT-PCR test. A safer way to test for changes in the lungs is via ultrasonography, because the evolution of the disease can be tracked to inform clinical decision-making. Ultrasounds still cannot detect lesions deep within the lung, in which case a CT scan would be needed. However, lung ultrasonography can be the first detection step because it does not use radiation and the detection would be repeatable with lower costs. These techniques should be used to evaluate treatment or as a diagnostic test in conjunction with the molecular tests. CT scans and ultrasounds require trained personnel and enclosed space, making it challenging to consider it as the main diagnostic test.

For rapid diagnosis, Abbott and LabCorp looked into mass-producing antibody tests [43]. These tests focus on testing for Immunoglobulin G (IgG) antibodies because its responses relate to adaptive immunity and have immunological memory of previous virus encounters. The presence of these IgG antibodies can identify what specific antigen it has a high affinity for, and the previous virus is encountered [44]. In the context of COVID-19, the testing provides information on whether that patient was previously infected and whether immunity was detected or not.

The Abbott antibody test (SARS-CoV-2 IgG assay) has shown 100% sensitivity and 99.9% specificity thus far [45]. The Abbott test finds whether the patient has IgG antibodies for COVID-19, which can stay for months to years after a person has recovered. One laboratory can run up to 14,000 tests per day. While Abbott previously released a COVID-19 molecular test and the ID Now rapid test, this serological test claims to be the most accurate [46]. The Abbott tests are running on the ARCHITECT^®^ i1000SR and i2000SR laboratory instruments with more than 2000 of these machines in different laboratories; this allows for Abbott to focus on the distribution of the tests and not the machinery on which to read them [47].

While Abbot is a frontrunner in the production of these antibody tests, other corporations are also researching antibody tests. For example, ARCpoint Labs has started to offer an antibody test in Monterey [48]. This test ranks the patient into one of three categories: no exposure, exposure, exposure, and recovery. Currently, due to the FDA’s EUA, any company can release these tests without testing for accuracy. The rate at which these tests come out is high, but consumers should still take the necessary precautions that are advised by the CDC [49].

### 2.2. Viral Transmission

**Figure 2:**
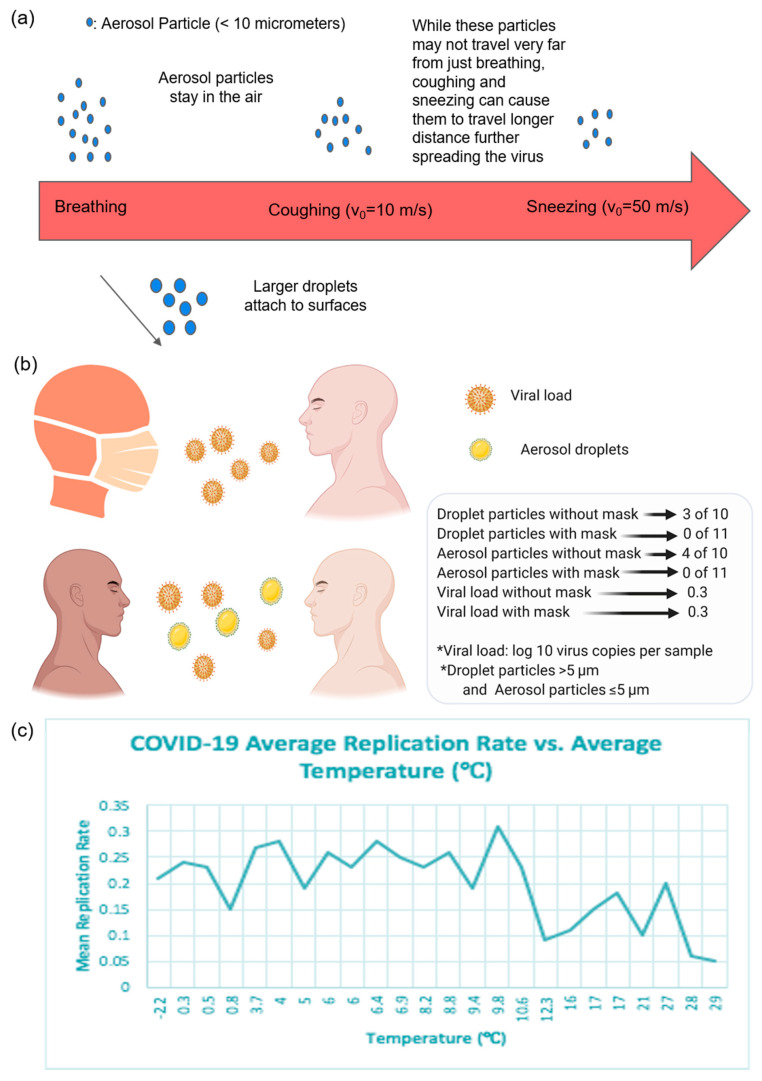
SARS-CoV-2 air travel, mask’s effect on transmission, and temperature dependence. (**a**) The spread of virus particles depending on coughing and sneezing, as well as particle size. The mode of ejection affects velocity and distance traveled, while the size affects the ability to stay in the air; (**b**) The difference in the spread of viral load and aerosol droplets with a mask and without a mask; (**c**) The relationship between the mean replication rate of SARS-CoV-2 and the temperature of 24 different countries is demonstrated. There is a moderate negative correlation between the mean replication rate and temperature, with a *p* value < 0.001. Data were taken from Caspi et al. [55] “Climate Effect on COVID-19 Spread Rate: An Online Surveillance Tool” with permission.

COVID-19 Transmission: The coronavirus spreads primarily through droplets that are generated by infected individuals during coughing, sneezing, or speaking. While it was initially believed that since these droplets are too heavy to stay in the air, the coronavirus is not airborne. Viruses may be airborne in the form of aerosols, which are fine particles that are able to stay in the air for a sustained amount of time. A recent study on aerosol and surface stability found that SARS-COV-2 was stable for three hours in its aerosol form [50]. This indicates that air transmission of the virus is possible, due to its capability to remain viable as an aerosol. While aerosolized viral particles cannot travel far from an infected patient breathing normally, coughing and sneezing can lead to traveling distances up to an estimated 20 feet [51]. These particles then remain suspended in the air near or far from their surface, depending on how the particles were produced, or fall out of the air and attach to surfaces in the case of larger particles (Figure 2a). Once these particles have attached to various surfaces, the length over which these particulates remain viable depends on the material of the surface. Under experimental conditions, it was found that SARS-CoV-2 and SARS-CoV-1 had similar stability times. For SARS-CoV-2, the half lifetimes are aerosol (1 h), copper (1 h), cardboard (3 h), stainless steel (5 h), and plastic (7 h).

Masks in COVID-19 Transmission: Individuals have resorted to wearing masks for protection against various viruses, but there is much controversy on whether these masks help. Masks might help keep people with COVID-19 from unknowingly transmitting the virus to others, and even though there is no harm in wearing masks, there is also no clear and significant benefit. When patients were asked to cough without masks, while wearing a disposable surgical mask and again wearing a 100% cotton mask, neither mask meaningfully decreased the viral load coughed onto the Petri dishes [52]. However, surgical masks reduced coronavirus detection in both droplets and aerosols. The virus was found in respiratory droplets in 3 out of 10 samples from participants not wearing masks, and in aerosols in 4 out of 10 samples taken without masks (Figure 2b) [53]. There was no detection of any virus in respiratory droplets or aerosols collected from participants wearing face masks that showed a trend toward reduced detection in respiratory droplets with the use of a mask. Thus, surgical masks reduced the droplets of the virus, but not the viral load that gets transmitted. To avoid spreading the virus before individuals experience symptoms, masks should be used in public venues.

Seasonal COVID-19 Transmission: Concerns have arisen as to whether a second wave of the coronavirus will return and whether the virus is seasonal. As societies start to lift lockdown regulations, social distancing rules must still be followed until the seasonality of the virus is confirmed. Current studies and previous research on SARS-CoV, influenza, and MERS-CoV shows that high temperatures and high humidity decrease the spread and transmission of the virus. For example, SARS is likely to stay on surfaces longer when the temperature is below 38 °C and humidity is below 95% [54]. On the other hand, influenza stays longer when the temperature is below 30 °C and the humidity is below 35%. This data suggests that COVID-19 is likely to occur during colder seasons, such as fall and winter, and stay longer in colder climates; these studies only show correlations between temperature and humidity as a function of mean viral replication rate (Figure 2c) [55]. Optimal temperature and humidity conditions of the survival of SARS-CoV-2 need to be validated over time in the year 2020 and beyond. After determining the presence of SARS-CoV-2 over all seasons, countries will be well-informed and prepared to enforce the necessary regulations.

### 2.3. Masks and Shields

With the spread of COVID-19, the demand for PPE grew as healthcare workers started following infectious disease protocols and as the individuals began shielding themselves from the virus. This necessity first emerged in Wuhan, China, which was met easily by domestic and international partners. However, as the pandemic spread into Europe and the Americas, it was evident that a shortage of PPE would be inevitable.

**Figure 3:**
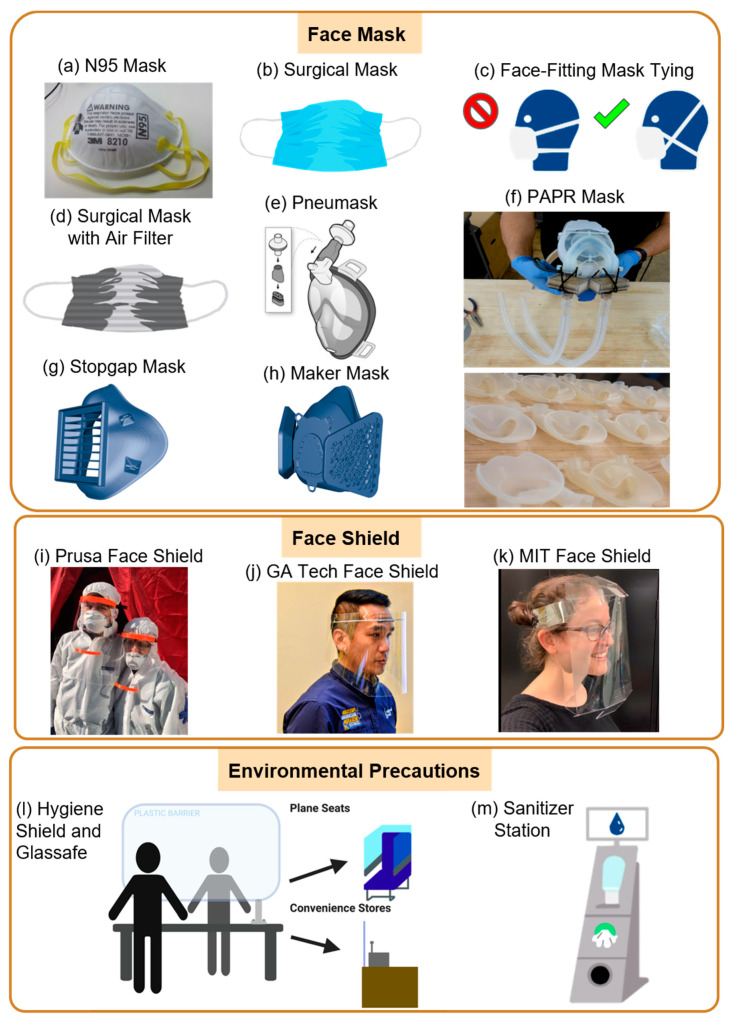
Protection tools against COVID-19 in the form of face masks, shields, and sanitizers. (**a**) The N95 Respirator is Food and Drug Administration (FDA)-approved and is currently the best-known form of personal protective equipment (PPE) available to healthcare professionals; (**b**) FDA-approved surgical masks are to be used by healthcare workers in the absence of N95 respirators; (**c**) The regular method for tying surgical masks compared to the newly proposed method that can secure the side openings of surgical masks; (**d**) A surgical mask design modified with filter performance rating (FPR) 9-10 air-conditioning filters; (**e**) The Stanford full-face, snorkel-inspired Pneumask. The Pneumask has deployed 2600 units all across the United States (US), with concentrations in New York, Massachusetts, and California; (**f**) The team at Duke has fully modified a powered air-purifying respirator (PAPR) design, which is attached to the surgical helmets (left) and the 3D-printed component of the PAPR (right); (**g**) The 3D printable Stopgap Mask from the Veterans Health Administration that is approved by the FDA to protect clinical personnel from COVID-19; (**h**) The 3D printable Maker Mask that can be used by non-clinical personnel when there are no FDA-approved masks available; (**i**) The Prusa Printer face shield that can be used to protect clinical personnel when no FDA-approved face shields are available; (**j**) The Georgia Tech 3D-printed rigid frame and a disposable face shield design; (**k**) The MIT origami disposable face shield. The Georgia Tech and MIT labs have collaborated to produce almost one million face shields to be used by hospitals around the US; (**l**) An illustration of the transparent acrylic barrier concept for public settings where cross-contamination is frequent. Aviointeriors has designed a possible solution for plane seats, while Safetell and Umdasch have designed and implemented their solution for retail stores; (**m**) The sanitizer station is a representation of the Umdasch Hygiene Station that could be distributed to all types of public facilities in the future as a norm.

Masks for COVID-19 Management: N95 masks (Figure 3a) were the first form of PPE to be at risk of running out in hospitals because of their ability to filter out at least 95% of airborne non-oil aerosols and smaller particle aerosols. These masks, made by the 3M Company, were approved by the National Institute for Occupational Safety and Health (NIOSH) and, when worn properly, are face-fitting to reduce the wearer’s chance of inadvertently inhaling virus particles. Naturally, Good Samaritan health centers, communities, and businesses responded to this PPE deficit. These innovators attempted to slow the spread of COVID-19 by modifying the original surgical mask design to mass-producing face shields around the world to develop environmental precautions for safe social distancing to ensue. For example, because surgical masks (Figure 3b) are not face-fitting, health professionals employed different tying strategies to secure the side openings (Figure 3c). Moreover, Wadhwani et al. created a method of improving surgical masks when an N95 respirator is unavailable [56]. Dr. Wadhwani attached air-conditioning filters with a filter performance rating (FPR) of 10 to the outer surface of regular surgical masks to increase the filtering ability of the mask (Figure 3d). The mask design filters out 95% of airborne particles larger than 0.3 microns, similar to N95 respirators. The mask was able to pass saccharin and smoke testing, but has yet to be tested with other FDA-approved regulations.

Self-Manufacturing Masks: The three-dimensional (3D) printing community has responded quickly and effectively. A community with over 2 million consumers of 3D printers and 140,000 industrial 3D printers around the world had the unique ability to change their item of production in hours instead of weeks and months, effectively creating elastic production capacity for any device that can be 3D-printed. Bioengineers at the Stanford Prakash Laboratory are currently developing the Pneumask, a modified full-face snorkel mask that can be reused by health professionals for PPE [57]. The Pneumask features one inhaling port at the top of the mask, a breathing circuit filter that filters air entering through the inhaling port, and two exhaling ports at the bottom of the face (Figure 3e). The eyes, nose, and mouth are sealed from outside contact in this full-face snorkel. The 3D-printed design can be disassembled and autoclaved for reuse. This product has not been certified as PPE yet. At Duke’s Innovation Co-Lab, bioengineers have created a powered air-purifying respirator (PAPR) design [58] to modify current surgical helmets (Figure 3f (upper)). Surgical helmets do not filter the air in the room, so the laboratory used 3D printing to create a filter that could be attached to the suits (Figure 3f (lower)). PAPR was tested by the Precision Air Technology for HEPA certification, and is currently used by Duke Health. This product has also not been certified as PPE yet. In addition to the face masks designed by the Prakash laboratory and the Duke’s Innovation Co-Lab, there are fully 3D printable designs from the Veterans Health Administration and a Maker Mask group in Seattle. Each mask design relies on filter patches to be inserted into the mask. The Maker Mask is only meant for non-clinical settings where there are no FDA-approved masks available (Figure 3g). However, the Stopgap mask from the Veterans Health Administration was approved by the FDA to protect clinical personnel from COVID-19 (Figure 3h). This approval was allowed as the mask had effective disposable filters, tightly fit around the nose and mouth, and was easy to disinfect.

High-efficiency particulate air (HEPA)-based air-conditioning filters are also a cause for concern. HEPA filters are effective in filtering particles larger than 0.3 microns that cannot pass through the filter, as well as smaller particles that bounce off other particles and cling to the filter [59]. HEPA filters would seem to be an adequate solution when an N95 respirator is unavailable, yet the dangers of using HEPA filters for face masks is that glass fibers in masks could be inhaled and cause throat and facial irritation unless the air is filtered properly.

With regard to homemade masks which the public has utilized for personal protection, hospitals are far less likely to utilize them in a hospital setting for any purpose other than for use by patients. This is due to their being constructed by a less effective material for blocking pathogens, and to them being made in non-medical-grade conditions that could cause contamination of the masks with microorganisms. Medical-quality masks are three times more effective in blocking the transmission of microorganisms than homemade masks, and are thus not suitable for use by medical professionals in the care of sick patients.

The new PPE and medical device designs that have emerged from the pandemic have not been through the extensive prototype testing that FDA-approved devices have been able to pass. It has not been confirmed as to whether the new devices effectively treat or harm users. The CDC has issued an official statement that recommends healthcare professionals to only use homemade masks (e.g., bandanas, scarves) for the care of patients with COVID-19 as a last resort, and focuses on maximizing current stores of PPE [60].

Most regulations regarding the donation of PPE stem from the Good Samaritan Drug and Medical Supply Donation Act [61]. This law puts into place the basis for donating medical supplies to hospitals and medical professionals. Those who donate medical supplies must be able to describe the conditions under which the equipment was made, so as to ensure medical-grade quality. Importantly, as long as the donator makes sure the medical group is aware of these conditions and does not withhold any information about them, the medical group and any patient may not be able to sue the donator for any injury or loss of life sustained while the donated equipment is being used.

However, using PPE that fails to comply with the FDA, NIOSH, and Occupational Safety and Health Administration (OSHA)-approved tools poses a threat to the health of healthcare and essential workers. To best filter aerosols and droplets, the FDA requires masks to pass the ASTM F 1862 fluid resistance test, the ASTM F 1215-89 particle challenge study, ASTM F2101-01 bacterial filtration efficiency (BFE), Mil- M369454C breathability, and CPSC CS-191-53 flammability [62]. The FDA cautions about the dangers of using 3D-printed PPE, and specifically 3D-printed masks and respirators, because while the PPE may provide a physical barrier, it might not be able to safely provide fluid resistance, bacterial filtration, and breathability. Moreover, 3D-printed PPE may not be face-fitting or function as properly as those manufactured by a medical company. Open-source 3D models from the same STL file, in theory, produce the same device, but in reality, these 3D files can result in an unexpected variation in the product, due to differences in g-code variables when translated across different devices. Likewise, different 3D printers have different thermoplastic filaments that vary in composition and properties. 3D-printed PPE can pose a possible threat if the filament is prone to retaining moisture in the environment, which can inadvertently transmit virus particles [63].

The Role of Face Shields: Covering the entire face is another highly sought-after PPE for healthcare professionals [64]. Although face shields are not a fool-proof method of protection against viral particles, a short-term barrier is sustained against the exposure of respiratory droplets by 97% after five minutes, and 81% after thirty minutes [65]. Within a week of COVID-19 being classified as a pandemic, one of the biggest leaders in the 3D printing community, Prusa Printers, put out a face shield model that was verified by the Ministry of Health in the Czech Republic for use in clinical settings (Figure 3i) [66]. Prusa Printers alone printed and assembled 55,000 face shields in three weeks [67]. This initial effort was the beginning of a wave of 3D printers that started printing face shields for their medical workers in the community, with over 250,000 downloads.

Researchers at the Georgia Institute of Technology (Georgia Tech) and Emory University worked together to design a DIY shield frame that can be mass-produced using injection molding (Figure 3j). The Georgia Tech team produced up to 2000 rigid frames per day, 3000 rigid shields per day, and 3000 disposable shields per day for hospitals all around Atlanta [68]. The designs were made available to others for worldwide manufacturing. Additionally, The Project Manus team at the Massachusetts Institute of Technology developed a single-sheet, disposable face-shield design that can be mass-produced using die-cutting (Figure 3k) [69]. The Project Manus team produced 50,000 shields per day to be shipped out to hospitals where healthcare professionals can easily store and assemble the disposable face shields. Teams at the University of Cambridge and the University of Queensland have also designed HappyShield, which is a curved-crease origami face-shield for infection control [70]. HappyShield can be easily assembled on-site and decontaminated for reuse over high numbers of shifts. These products from different teams have not yet been certified as a PPE.

The Good Samaritans have taken yet another approach to provide solutions for the world’s hospitals in their time of need. Volunteers put together their sewing efforts to donate DIY cloth face masks to hospitals, healthcare facilities, and nursing homes. The CDC has released an official guideline on how, when, and where cloth face masks should be used, as well as the instructions for the public on how to make cloth face masks. Specifically, the CDC recommends the public to use tightly woven cotton for their homemade masks to slow the spread of the virus in public places.

Workplace Shields for Prevention: Another population besides healthcare professionals at risk of contracting COVID-19 and spreading the virus is essential workers. Financial institutions, retail stores, supermarkets, manufacturing plants, and funeral homes are all essential businesses under stress due to constant social interaction, proximity to others, and extended exposure from the high number of shifts. Safetell, a physical security solution provider, has designed the Hygiene Shield, which allows staff to continue communicating safely to minimize physical contact (Figure 3l). The clear glass wall can be easily disinfected and protects both consumers and employees from spreading the virus. Hygiene Shields can be used for essential businesses that require the transaction of items from consumer to employee, and vice versa.

Umdasch, a German company that creates special retail solutions, has developed various devices to support retailers in the pandemic. Similar to Safetell’s Hygiene Shields, Staff Protection Shields are acrylic glass shields that allow employees to communicate effectively while maintaining social distancing (Figure 3l) [71].

Aviointeriors, an aircraft interior and passenger seat producer, designed the Glassafe and the Janus Seat to remodel aircraft seating arrangements in response to COVID-19 [72,73]. Glassafe is a transparent glass covering that can be installed on existing seats to isolate the passenger on the sides and back of the seat (Figure 3l). While the Janus Seat’s material is similar to that of Glassafe, the Glassafe configuration allows passengers in the center to face backward, instead of all passengers facing the flight direction. This solution isolates all three passengers from each other, and at the same time, the rows in front and behind them and people who walk through the aisle are socially distanced.

The Hygiene Station has a sensor-based disinfectant dispenser for contactless hand disinfectant, a disinfectant wipe dispenser for sanitizing baskets and trolleys, and an optional face mask dispenser. The Hygiene Station also has a customer enumeration strategy for access management by controlling numbers of customers in the store at a time to enhance social distancing measures (Figure 3m).

### 2.4. Gloves

**Figure 4:**
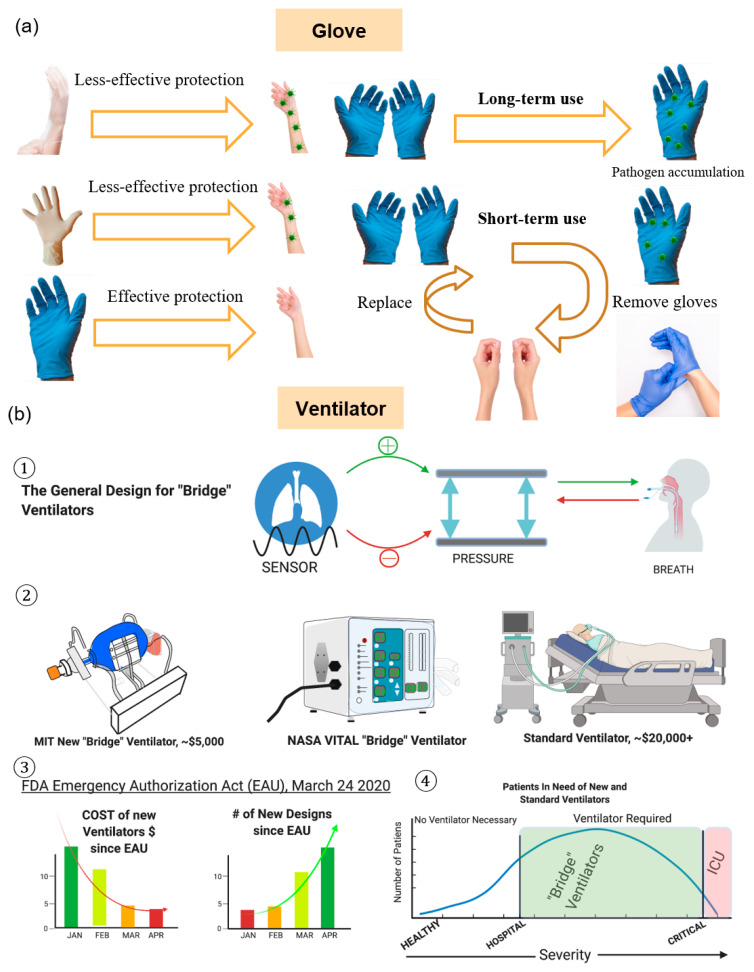
COVID-19 control by gloves and ventilators. (**a**) Gloves must be changed regularly and properly disposed of in biosafe disposal units. Not all gloves block the penetration of the virus. Thicker gloves offer a thicker barrier and better defense against COVID-19 droplets; (**b**) (1) The general design for new ventilators includes a breath sensor that senses the timing and size of breaths, a pressure pump that pumps air into and out of the lungs, and a tracheal tube that pumps air directly into the lungs; (2) Depiction of the Massachusetts Institute of Technology (MIT) New “Bridge” ventilator which uses a mechanical pump and a bag-valve-mask to administer air (left). The National Aeronautics and Space Administration (NASA) VITAL uses sensors and a mechanical pump to deliver safe breaths. Their design is licensable to accepted manufacturers (middle). A standard intensive care unit (ICU) ventilator is fully equipped with vitals monitoring, air delivery, and a computer that measures vitals and pressure for safe breathing. The safest option for patients in critical condition but are more expensive to produce are presented (right); (3) The Food and Drug Administration (FDA)’s Emergency Use Authorization (EUA) for mechanical ventilators helped drastically reduce the cost of new devices and increased the number of new device designs; (4) Newly developed ventilators are termed “bridge” ventilators and are intended for an intermediate use between hospitalized patients and critically ill patients. “Bridge” ventilators are used when standard ICU ventilators are not available, and standard ICU ventilators are still the safest option for patients in critical condition.

Gloves have long been used as a means to ensure the safety and wellbeing of healthcare workers who are at risk of being exposed to deadly pathogens. However, with the advent of the novel coronavirus, many have wondered whether or not the usage of gloves, in both medical and non-medical grades, can help prevent the spread of the virus. For medical purposes, the CDC recommends that all personnel directly involved in the care of patients with COVID-19 wear medical-grade gloves at all times, and frequently change them to prevent the accidental spreading of the virus (Figure 4a). Without proper protocol, gloves in a medical setting can carry microorganisms and will lead to increased rates of infection among both patients and medical professionals.

For non-medical settings, the CDC does not recommend the usage of gloves for containing the spread of COVID-19, unless caring for an infected family member where there is a risk of being exposed to bodily fluids. While the gloves themselves have been proven to be effective, many individuals without medical training are far less likely to frequently replace them, and thus risk contaminating others with dirty gloves. Instead, the CDC recommends frequent use of hand sanitizer containing 60% or more alcohol, or frequent handwashing with soap and water for at least 30 s, to ensure any microorganisms are cleaned off [74].

For medical professionals using gloves as a PPE, only certain materials are approved by the FDA for use as medical-standard materials for use in glove production (Figure 4a). For routine inspections that are not expected to bring a medical professional into contact with bodily fluids, latex and vinyl gloves are acceptable as a means for protection. However, for surgical settings or for situations where high exposure to COVID-19 is anticipated, thicker nitrile gloves are recommended and are considered to be the best option as a form of PPE [75].

### 2.5. Ventilators

Mechanical ventilators (MV) are mechanical breathing assistance devices that use mechanical pressure to pump air into and out of the lungs. These devices are used when patients are not able to breathe independently. During the COVID-19 surge, the need for MV is exceeding capacity and mechanical engineering teams from the public and private sector have started to rapidly manufacture new devices.

The general design for MV includes an adjustable air pump, a breathing monitor, and an exhalation valve. Air administration may use either a face mask that covers the mouth, or an intubation tube that flows air directly into the lungs (Figure 4(b1)). Pressure sensors and air volume sensors monitor breathing and time the air-pump cycle and breath volume. How long a patient uses the vent and weaning a patient off is determined by careful tests conducted by physicians [76].

As the demand for low-cost, compact, and sanitary ventilators are rising, teams of engineers from universities and the private sector are making new designs (Figure 4(b2)). A Stanford team developed a $1500.00 device, which was donated to hospitals in Peru [77]. MIT developed a prototype based on a design from a mechanical engineering class in 2010 [78]. The team partnered with the New York City Economic Development Corporation to supply 3000 devices to the New York City hospitals [79]. A Georgia Tech team, led by Dr. Shannon Yee, partnered with the Emory Healthcare system to design a prototype [80]. Their design is an adaptation of bag-valve-mask resuscitators, with manual tidal volume adjustment and dual patient usage. A team of both NASA and California Institute of Technology engineers developed a device named the Ventilator Intervention Technology Administered Locally (VITAL) [81]. Their device is capable of handling outdoor terrains, and has a device sterilization procedure to sterilize between patients. The device was developed in only 37 days. The low-cost devices are termed “bridge” ventilators or automatic resuscitators that are meant to be used before a critically ill patient is put on a “standard” ventilator (Figure 4(b3)). FDA approval processes of the NASA’s VITAL ventilator and the MIT’s “bridge” ventilator were both expedited by the FDA’s EUA.

The FDA employed the EUA as of 24 March 2020 for ventilators to fast-track new approvals for designs that are not currently legally manufactured (Figure 4(b4)). The Act states the FDA will aid in the development and distribution of the newly approved devices. Approval reduces the number of requirements for the device. The Act only approves the ventilators for the duration of the COVID-19 pandemic, while the EUA is in effect. The FDA’s aid and the global need for ventilators have decreased the cost of manufacturing ventilators and increased the number of new designs. Medtronic already had a compact, lightweight, and portable ventilator that is termed the Puritan Bennett 560. The company plans on introducing the device in the USA under the EUA, and to ramp up production to 25,000 units by the end of the summer. Medtronic, the MIT team, and NASA are all licensing their blueprints for free or at a reduced cost to other manufacturers.

The use of mechanical ventilators on high-risk patients over the age of 65 years is being put under scrutiny. Based on a new report of 5700 patients hospitalized with COVID-19, of the patients between 18–65 years old, 76.4% died on mechanical ventilation, and 19.8% of patients died who were not on mechanical ventilation. Of the patients older than 65 years, 97.2% died on mechanical ventilation, and 26.6% died who were not on mechanical ventilation [82]. The discrepancy between patients who die on and off mechanical ventilation warrants further examination into the risk factors associated with using mechanical ventilation in different patient age-groups, disease stages, and disease severities.

### 2.6. Machine Learning Analysis of COVID-19

Machine Learning (ML) advances can be helpful in many aspects of the COVID-19 pandemic, such as molecular, clinical, and societal utilization.

**Figure 5:**
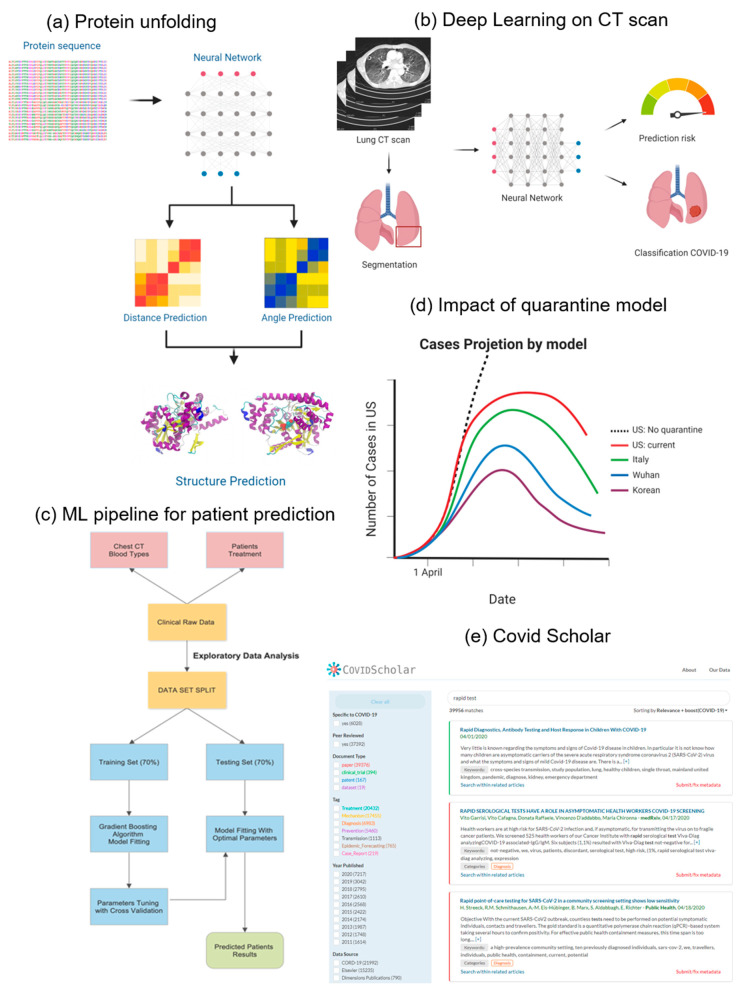
Machine learning analysis of COVID-19 dynamics. (**a**) Protein unfolding prediction. The first step is a multiple sequence alignment that compares a protein’s sequence with a similar one, suggesting a close location in the folded protein. The neural network learns to accurately predict the distance by training on precisely measured distances in proteins. In parallel, another neural network is trained to predict the angles of the joints in the folded protein chain. Finally, gradient descent is used to find a physically possible folding of a protein; (**b**) A deep-learning framework on a CT scan. First, the lung region is extracted using a segmentation method. Then, the corresponding image of the lung region is passed onto the deep-learning model that predicts the risk value based on the CT scan; (**c**) Machine Learning pipeline for patient risk forecasting; (**d**) Pandemic forecasting using machine learning under various quarantine rules. The model predicted the infected case count of the US with quarantine control and the exponential increase of cases if there were no quarantine measures. It also shows predicted case counts under various quarantine measures, as implemented in Wuhan, Italy, and South Korea. All of them led to a plateau of cases sooner than the US model; (**e**) COVID Scholar website for scientific article search. Searching for the term “rapid test” gives all journal articles related to the term. This can be further filtered depending on the year, the type of document, whether they were peer-reviewed, and whether they are specific to COVID-19.

Protein structure prediction for COVID-19 Drug Design: From a molecular perspective, ML models can be used to better understand the proteins that are involved in SARS-CoV-2 infection to help the search for potential targets for treatment. First, an AlphaFold [83] model was used to predict the Protein 3D structure that was determined by amino acid sequences and their influence on the role and function of the protein of interest [84] (Figure 5a). Biomedical data reconstructs the relationships between proteins and drugs that can predict potentially effective drug candidates [85]. Therefore, at a molecular scale, ML has the potential to predict the structure of the protein, identify existing drugs for targeting these proteins, and enable COVID-19 treatments.

Large-Scale Analysis of COVID-19 CT Scans: From a clinical perspective, ML can be used to detect COVID-19 and predict patient outcomes. Pathogenic laboratory testing is the standard for screening suspected cases, but this process is time-consuming, with significant false-negative results. Therefore, alternative diagnostic methods that are both accurate and rapid to combat the disease using machine learning to support diagnosis from CT scans are needed. This is because of the virus exhibiting radiological signs and image patterns that can be observed in CT scans, but identifying these signs remain difficult and time-consuming, even for radiologists. Therefore, because of the complexity of the imaging data, researchers developed a deep-learning-based model for automatic screening and diagnosis of COVID-19 detection, based on CT chest scans [86]. The inputs are pre-processed CT pictures from which the model first extracts a region of interest (ROI) before passing it on to a convolutional neural network that classifies each ROI using ensemble methods. Three-dimensional CT volumes can be used to detect COVID-19 and its transmission patterns [87]. A deep-learning-based algorithm takes a lung-region 3D CT scan and segments it using a pre-trained U-net neural network. Then, the segmented region is fed into a 3D deep neural network and predicts the infection rate. Taking 3D space information for a weakly-supervised deep-learning model improved the prediction for infection (Figure 5b).

Pandemic Predictions for Hospitalizations: To prepare, plan, and optimize the health system during the pandemic, forecasting patient outcomes is critical. Factors that make patients at risk for hospitalization should be identified to calculate the probability of developing acute respiratory distress and organ failure during clinic treatments. Based on the dataset that includes blood samples and CT scans from patients, machine learning models can identify patients at high risk and who might develop respiratory distress in the future. Using models such as Gradient Boosting or the Support Vector Machine, key measurable and explainable features that influence those risks can be determined (Figure 5c).

Spatial-Temporal Dynamics of Pandemics: From a societal perspective, ML can be used to predict the number of cases under various quarantine policies and cluster geographical regions to find similarities. Understanding how the virus is transmitted, and its effect on different demographics and geographic regions is crucial for public policy healthcare interventions. The effect of quarantine control in the COVID-19 spread can be accurately predicted using deep learning (Figure 5d). A data-driven deep-learning model was developed to interpret and extrapolate publicly available data that was computed with the first-principles epidemiological equations [88]. Then, isolation measures in different countries and their effects on controlling the reproduction number of the virus were compared. By using this model, the spread of the virus to implement reasonable quarantine rules could be predicted. An autoencoder was created for real-time forecasting of new cases using the latent variable layers to extract the most important features for each geographical region. The autoencoder was fed into a k-means clustering algorithm to group similar regions for further analysis [89].

COVID-19 Literature Management: Finally, other machine learning tools developed can indirectly help researchers and the public fight the pandemic. Covidscholar is a text-mining website aimed at synthesizing a huge amount of scientific literature on COVID-19 using neuro-linguistic programming (NLP) to automate search. This is helpful because more than 200 new journal articles are being published every day on the coronavirus. A simple search for a “rapid test” in Covidscholar provided organized and classified data that can greatly expedite COVID-19 research (Figure 5e).

### 2.7. Pandemic Simulations

**Figure 6:**
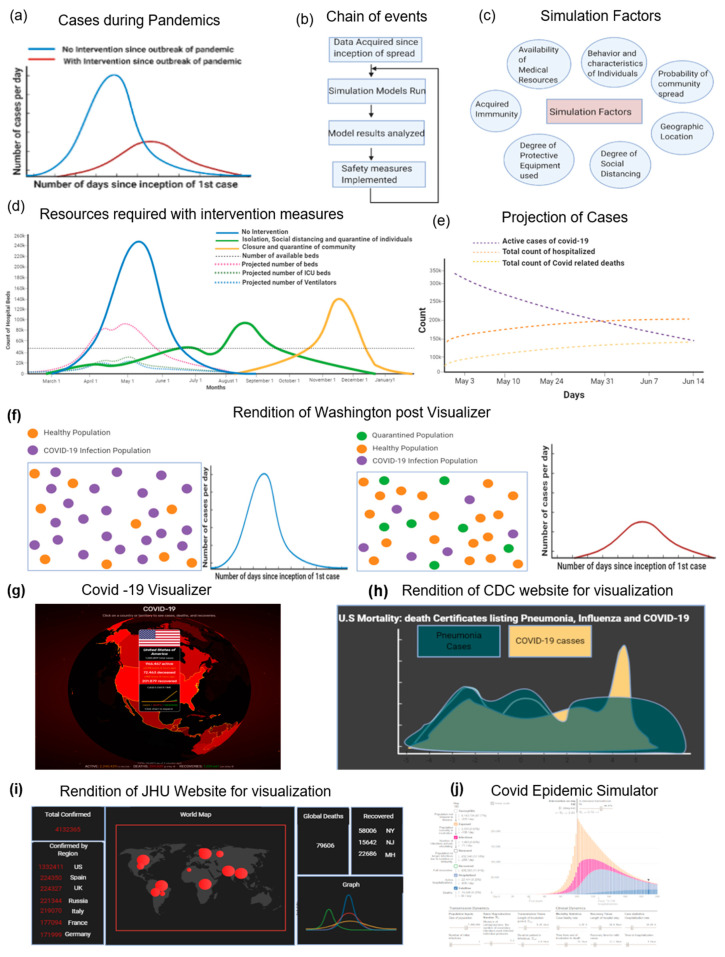
Simulation tools for COVID-19 pandemic modeling and predictions. (**a**) Comparison of epidemic curves with (red) and without (blue) intervention, as suggested by the CDC; (**b**) The sequence of events initiated by utilizing the results of a simulator is shown; (**c**) The main factors considered for the COVID-19 simulators, such as measures of social distancing and probability of community spread; (**d**) Comparison of projected curves resulting from various simulations that cover different scenarios of social distancing, for the months of March to January in the US; (**e**) Projection of the number of active cases, those hospitalized, and deaths from COVID-19, simulated between May 3 to May 31 in the US; (**f**) A rendition of the billiards balls model by the Washington Post, to illustrate the benefits of social distancing. (LEFT) Simulation rendition which depicts effects caused if social distancing is not followed. (RIGHT) Simulation rendition, if social distancing is followed. The simulation renditions are followed by epidemic curve graphs; (**g**) A platform provided by the COVD-19 Visualizer website, which updates the number of cases worldwide in real-time; (**h**) A rendition of the comparison of mortality cases related to COVID-19, influenza, and pneumonia in the US, as visualized by the CDC; (**i**) A rendition of the various visualizations on COVID-19 spread worldwide, provided by the Johns Hopkins website; (**j**) Screenshot of an interactive simulator involving various metrics that predicts the severity of COVID-19 spread.

COVID-19 Data Collection: Since the outbreak of COVID-19, WHO and most countries around the world have been trying to overcome the disease over the past few months by extensive testing and simulations that predict the degree to which the world would be affected. This is achieved by the data collected by healthcare and government institutions around the world. The simulation models have multiple sources of data that come from the WHO, National Health Commission of the People’s Republic of China (NHC), the COVID Tracking Project, European Centre for Disease Prevention and Control (ECDC), and CDC. Notable websites on pandemic data include 1point3acres, Worldometers.info, and BNO news. COVID-19 is prone to community transmission, and is therefore harder to track and to control its spread. Epidemic curves are often plotted in an attempt to understand the extent of infections and the implications of preventative measures. The CDC suggested that adhering to strict preventative measures would make it possible to flatten the epidemic curve, which is illustrated by a cartoon rendition (Figure 6a). The simulation models are implemented by taking the current level of threat that is posed to society into consideration, as well as the adverseness of the measures that need to be taken. The results of the models are usually analyzed by governing institutions, while implementing appropriate measures such that it minimizes economic and financial distress, as depicted in the flowchart (Figure 6b). To get accurate results from the simulation models, researchers need the appropriate information and resources to make their models work (Figure 6c).

Hospital Resource Predictions: The Institute for Disease Modeling produced software called Epidemiological Modeling (EMOD) [90], which is used extensively by epidemiologists and research groups to understand the effects of taking measures to eradicate the disease, or at least slow it down. Utilizing mathematical basis and concepts, factors such as susceptibility to disease, cases which tested positive for COVID-19, and cases that recovered from COVID-19 are included. There are also assumptions made regarding the probability of a person catching COVID-19 in a population, which is further divided into groups to understand the propagation of the disease. The depletion of healthcare resources, such as the number of beds available in the US, can be circumvented by taking appropriate social distance measures. Simulation models predict the availability of the number of ICU beds, regular hospital beds, and equipment that are required, such as ventilators (Figure 6d).

Social Pattern Analysis: Several of the other common models deal with scenarios of the entirety of the population at the outset (single group model), such as social hierarchy, division of the existing population into subpopulations (composite group model), characteristics and traits of individuals (individual models), and so on. For COVID-19 models, a model that capitalizes on individual traits and behaviors can be accurately simulated. To construct the model, a set number of rubrics about the behavior of individuals are considered. For example, the set of actions and time of execution of actions for each individual would be rigid. Given the prior movements of the individual, it should be possible to fully predict the individual’s whereabouts. Of course, except in cases such as the “Diamond Princess”, this would hardly be an accurate assumption, as it does not reflect the reality of the situation. If the models need to consider extensive individualistic behavior, to make them as accurate as possible, there is a need to acquire a huge amount of data. The active number of cases is predicted to decrease steadily until mid-June (Figure 6e). The “Diamond Princess” ship is an interesting case study, as it was in controlled settings and optimal for parameter control. This case study helps in the analysis of how prevention of the COVID-19 infection could have been brought about by implementing control measures. Based on the environment that exists on the “Diamond Princess” cruise ship, a disease transmission model based on crowd flow control would be feasible for simulation purposes.

There are several simplified online simulators available to the general public to experiment with and gain an understanding of the importance of social distancing to minimize the damage caused. The Washington post also simulated a COVID-19 model based on a billiard ball model that conceptualizes many spherical structures that locomote within a bounded rectangular region, of which a rendition is displayed (Figure 6f) [91]. This model is simplistic and is mainly used to spread awareness of the disease. Agent-based models can be used for the analysis of bias-variance trade-offs, probabilistic graphical models, differential equations, and game theory [92]. Apart from simulations, multiple websites, such as the COVID-19 Visualizer, used to update people on the number of cases worldwide in real-time were developed (Figure 6g). The CDC website helps visualize multiple statistics and graphs for the pandemic’s dynamics (Figure 6h). A website interface that is hosted by Johns Hopkins University provides various visualizations of the spread of COVID-19 (Figure 6i). More complex models include interactive Python scripts to quantify the degree of social distancing, geographic areas, and ease of movement, such as a COVID-19 epidemic simulator (Figure 6j).

Computational Resources for COVID-19 Simulations: Another set of simulations in the fight against COVID-19 involves running calculations in epidemiology, bioinformatics, and molecular modeling to study the virus’ spread and its dynamics. High-performance computing and distributed computing are a few approaches that have been demonstrated to run experiments that would take months or years to complete on a traditional computing platform at a faster pace. The COVID-19 High-Performance Computing (HPC) Consortium combines the computing capabilities of advanced and powerful computers to allow researchers to run projects related to the disease which require high computing power [93]. This consortium includes a range of computers from small clusters to supercomputers from the U.S. Department of Energy national laboratories, companies like IBM, Google Cloud and Amazon Web Services, federal agencies such as NASA and National Science Foundation (NSF), and various academic institutions, such as the Massachusetts Institute of Technology and the University of Texas at Austin. Currently, there are 36 active projects on topics such as social modeling and forecasting based on epidemiology models, understanding the structure of the virus at the molecular level, analyzing the viral RNA, studying the DNA of the patients infected, and suchlike. As an example of the potential of this approach, using the world’s most powerful supercomputer Summit built by IBM, researchers were able to simulate more than 8000 small-molecule medications and natural product compounds within days and identified 77 compounds that had the potential to bind to the virus’ spike protein [94]. Another project that uses HPC is COVID MoonShot, by PostEra, a start-up company linked to Cambridge University. This project is a crowdsourcing initiative that accepts submissions of designs of potential drugs from chemists and uses machine learning to screen them and select the most promising candidates for synthesis and testing [95].

Folding@Home is a distributed computing project based at Washington University in the St. Louis School of Medicine [96]. It focuses on disease research, connecting the unused processing power of laptops and desktop computers around the world to create a virtual supercomputer. This project aims to understand how the spike protein in SARS-CoV-2 works and how therapeutics can be designed to stop it from infecting host cells, using computer simulations to understand the protein’s folding process, considering the structures obtained from experiments as the starting point. These simulations yield the structures that are otherwise hidden and identify potential sites to act as drug targets. Such a simulation has shown to be useful in the case of the Ebola virus, uncovering a previously unknown structure of the viral protein with a druggable site [97]. Rosetta@Home is another such distributed computing project, run by the Baker laboratory at the University of Washington, to predict the three-dimensional shapes of the viral proteins in their final folded state [98]. Unlike Folding@Home which models the nature of protein folding, Rosetta studies only the final protein structure. These projects can help access the structure and function of the proteins in the fight against the virus.

### 2.8. Digital Monitoring

**Figure 7:**
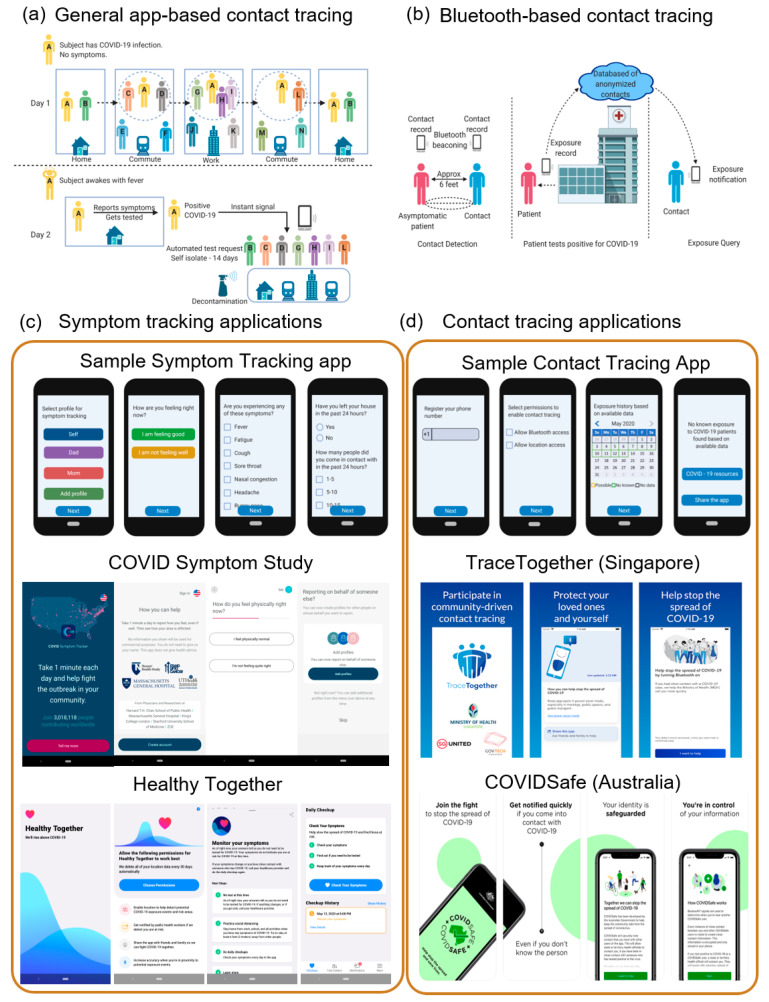
Digital monitoring tools of COVID-19 spread by online and cellphone applications. (**a**) General smartphone app-based contact tracing—the social encounters (distance less than 6 feet) of all the users are recorded by the app, using methods such as GPS information, QR code-tracking, and Bluetooth communication. An asymptomatic patient could infect others in their family, commute, or workplace. The patient takes the COVID-19 test (using the app) when individuals show signs of symptoms, such as fever. When the patient tests positive, instant notifications are sent to individuals who could be infected by the virus. Individuals are asked to take the COVID-19 test and self-isolate themselves for 14 days. The places where the patient went before testing positive are also thoroughly disinfected to prevent the spread of the disease; (**b**) Bluetooth-based contact tracing, as proposed by the MIT Lincoln Laboratory, does not require personal information or location to be shared. The app keeps a record of Bluetooth signals it has encountered, along with information about the distance between the devices and duration for which other individuals were nearby. When a user tests positive, the app sends the contact record to the database of anonymized contacts, which then sends exposure notifications to the users who might have been in close contact with the patient. This app works in a privacy-sensitive manner, without revealing user information to other individuals and health authorities; (**c**) Examples of apps that use surveys to track the symptoms of users to monitor the prevalence of the disease and identify potential hotspots: screenshots from a generic symptom tracking app to show sample survey questions, COVID Symptom Study (by Massachusetts General Hospital, the Harvard T.H. Chan School of Public Health, King’s College London and Stanford University School of Medicine, working with ZOE—a health science company) and the Healthy Together (by State of Utah, USA); (**d**) Examples of contact tracing apps: screenshots from a generic contact tracing app to show user interface and official government-developed apps—TraceTogether (Singapore) and COVIDSafe (Australia). These apps use Bluetooth-based contact tracing and provide region-specific healthcare guidelines and information.

Social Contract Tracing: In the past few months, many countries have gone under lockdown to slow down the transmission of the virus. However, since lockdown disrupts productivity and affects the livelihood of the people, an approach called contact tracing has been implemented in many places to contain the disease while allowing people to live a new normal [99]. Contact tracing is a way of identifying potentially infected people by studying people’s social interactions [100]. It helps to notify healthcare workers of potential cases and the people of their risk of infection. The government can use this information to recognize infection hotspots and come up with strategies. This process is an important step that has been used in the past to slow down epidemics such as Ebola and HIV. While manual contact tracing is tedious and unreliable, an automated digital approach using smartphone apps, device tracking data, and surveys may be a better solution [101]. A general approach to smartphone app-based contact tracing is illustrated in Figure 7a. Apart from apps, a global positioning system (GPS) and quick response code (QR-code)-based wristbands and bracelets are also used for tracking and enforcing quarantine so that authorities are alerted if a patient leaves their designated area.

Smartphone-based COVID Monitoring: There are many smartphone applications developed and deployed around the world, with a few of them being supported by the official government in countries like China, Singapore, and India. Different approaches have been taken by different countries; for example, many countries require citizens to enter their personal information and allow location tracking, while others use law enforcement monitoring authorities to automatically track the movement of citizens. Most COVID-19 contact tracing applications use Bluetooth to locate phones nearby that use the same application (Figure 7b). The apps then keep a record of the Bluetooth signals individuals encounter in distinct locations. These Bluetooth signals can contain information about the distance between people and the number of times where individuals were close to each other. When a person tests positive, the result is uploaded to the app which then notifies other people who may have been exposed to the virus, based on how close individuals were to the person.

Apart from contact tracing and alerting, several of these apps also help in tracking symptoms, self-diagnosis, and providing healthcare information to the users [102,103,104,105,106]. Apps like the COVID Symptom Study [107] and HowWeFeel [108] by the T.H. Chan School of Public Health, and web-based surveys like the COVID-19 Screening Tool by Apple and CDC, COVID-19 Symptoms and Social Distancing Web Survey, and Global COVID-19 Survey by Harvard T.H. Chan School of Public Health have daily surveys that help in collecting data about the prevalence of COVID-19 symptoms in a location-based, anonymous manner (Figure 7c) [109]. These surveys collect details such as demographic background, zip code, health conditions, daily symptoms, possible exposure to others with COVID-19, and social distancing behavior. This data can help health agencies to predict virus hotspots and respond better to the pandemic.

One of the key concerns regarding using a smartphone app for contact tracing is the issue of privacy [110]. Individuals are reluctant to share their private information with other individuals, public health professionals, the government, or cell service providers. This issue has been considered and there are a few privacy-sensitive apps, such as TraceTogether [111], an app used in Singapore, and CovidSafe [112], an app developed by the University of Washington and Microsoft, which uses Bluetooth communications between devices to track contact (Figure 7d). Similar efforts have been led by researchers at MIT, who came up with a smartphone-assisted contact tracing system, called Private Automated Contact Tracing (PACT) [113], which identifies people who are at risk of COVID-19 infection in an anonymous and location-independent manner by using Bluetooth communication between cell phones in proximity. While the technology has been tested at the laboratory, large-scale use has yet to be implemented. A major initiative in this area is the Google and Apple contact tracing project which combines Bluetooth Low Energy technology with privacy-preserving cryptography to create an exposure notification system. Being a collaborative effort between Google and Apple, the Exposure Notification application programming interface (API) works across both Android and iOS devices. Using the API, app developers working on behalf of public health authorities can build applications for smartphones according to their specifications. In the future, there will be an integration of this system with Google’s Android and Apple’s iOS operating systems to rely less on apps.

Online Search Method for COVID-19 Tracking: Another method of digitally monitoring the spread of the disease involves studying online search behavior [114]. A study by researchers from University College London, in collaboration with Public Health England, Microsoft Research, and Harvard Medical School, uses machine learning to track COVID-19 by using keywords from Google search queries and COVID-19-related news coverage daily. Google search data is collected from the Google Health Trends API. It includes queries about COVID-19-related symptoms, as identified by the NHS FF100 survey, and other coronavirus-related terms. Their current model uses online search frequency time-series to predict the prevalence of COVID-19 in a few countries, including the United Kingdom, the United States of America (USA), Australia, Canada, and Italy. This research can help identify potential positive cases who are otherwise not tested and help understand the true extent of community spread.

## 3. Discussions

### 3.1. Vaccine Regulations

As of now, vaccines are under development to prevent COVID-19 in healthy individuals. A vaccine has an integral role in protecting society and removing current social distancing protocols. Currently, multiple pharmaceutical companies and university labs are working on the creation of the vaccine in collaboration or independently. These partnerships are utilizing existing vaccine technologies that include inactivated viruses, virus-like particles, protein nanoparticles in matrix-M, non-replicating or replicating viral vectors, RNA- or DNA-based vaccines, and protein subunits. Along with researching the platform for the vaccines, different routes of administration are useful in the development of these vaccines. With the four phases of clinical trials necessary for FDA approval, COVID-19 vaccines are projected to be available to the public in 12–18 months [115]. Tracked by the Regulatory Focus Professionals Society, a few of the most promising vaccines under clinical trials are an mRNA vaccine and a non-replicating viral vector vaccine [116].

The University of Oxford is using a non-replicating viral vector from chimpanzees’ adenovirus to target the S glycoprotein, which is under a Phase I clinical trial [117]. The vaccine contains the weakened version of the adenovirus with the genetic material of the SARS-CoV-2 spike protein. This spike protein acts as a protection for the immune system if COVID-19 infects the body. The University of Oxford and AstraZeneca have also paired together for the global development of this vaccine following the clinical trials [118]. Current estimates dictate that if human trials are successful, then this vaccine may be publicly available by September [119]. Vaccine development is typically a long and expensive process, with high attrition and stringent involvement of the FDA. However, in the case of a pandemic, many steps, such as clinical development and manufacturing development are done parallelly to speed up the development process. Previous tried-and-true methods must be utilized in vaccine development to allow for sufficient manufacturing [120].

### 3.2. Testing Regulations

To further aid in terminating this pandemic, a global testing strategy is needed such that a significant portion of the population gets screened for the disease. Doing so ensures that we have a better understanding of the disease progression and can further help in allocating medical staff and resources in different hospitals, cities, and countries. Currently, there are more than 20 different test kits for COVID-19, each with a different rate of producing false positives and/or false negatives [121]. Furthermore, countries have varying guidelines for reporting infected cases. These factors increase the variability in the reported data and are uninformative about the disease progression, which negatively affects several nations’ ability to adequately prepare for this pandemic as it develops quickly worldwide. Therefore, countries need to have detailed testing guidelines and report their testing results regularly and openly.

Molecular testing checks for the presence of SARS-CoV-2 genetic material from respiratory specimens, either through swapping nostrils or sampling saliva. In addition to this, COVID-19 diagnostics involves serological testing for the presence of antibodies against SARS-CoV-2, and it can be done through a blood sample from a simple finger prick. Although serological tests are typically faster than molecular testing, a higher rate of producing false negatives is obtained because the human body starts producing antibodies against the virus a few days to a week after the infection. What further increases the complexity of the problem is the fact that the FDA has approved over 40 serological tests from different manufacturers, each with different precision and accuracy measures [122].

### 3.3. Immunity Ethics

Several countries are considering giving an immunity passport via antibody test results in place of a vaccination certificate to identify which individuals are ready to go back into society. An immunity passport would allow individuals to return to their workplaces and schools, as well as other aspects of daily activities; it could act as the first step in society returning to its status quo. There is a gap in understanding the time frame for which IgG antibodies last, as well as the number of antibodies needed to prevent infection, which would be critical to assigning citizens the immunity passport. Antibody tests still need to have acceptable specificities and sensitivities, as well as optimize false negatives and positives. If an individual is false-positive and gets an immunity passport, then the individual could unknowingly infect others. These passports cannot be given based on self-reporting of COVID-19 either just because a couple of individuals did not get tested. With these current gaps in knowledge, the antibody test field needs to be researched further before an immunity passport becomes an adopted policy.

Another topic to consider is which careers and places would need this immunity passport. Places of large public gatherings such as restaurants, clubs, and public transportation systems would have to check for it. Healthcare professionals, restaurant staff, and other jobs involving human contact would require this immunity passport. An issue that arises with the assignment of an immunity passport is that it excludes several workers and is liable to corruption, as well as social inequities. Particular people would have better access to these antibody tests and be able to prioritize their applications ahead of others. Additionally, many legal issues ensue in terms of whether the lack of immunity counts as disability and anti-discrimination laws. Vaccine certificates incentivize people to get vaccines, a beneficial act for the individual and society. On the other hand, these immunity passports seem to incentivize COVID-19 infection, which is detrimental to society. Countries that suggest using immunity passports should consider the societal consequences of this decision [123].

### 3.4. Healthcare Impact

A robust healthcare workforce is essential in responding to this pandemic. While unemployment is quickly rising in other industries with many businesses shutting down, more healthcare workers are still needed to deal with the sudden influx of patients. Many primary care doctors are working remotely via telemedicine and can address any patient’s concerns without risking the patient’s exposure to COVID-19. Previously, many physicians and patients resisted this concept of virtual healthcare since in-person consultations are vital to both parties. However, this virtual healthcare system allows physicians to see up to 100 patients per day and allows patients to avoid the long waiting-room time [124].

Not all patients can be evaluated by telemedicine. With the rise of COVID-19 cases, there is a need for healthcare professionals in hospitals and clinics to provide services in environments with high viral exposure, coupled with a lack of PPE. Most countries have a shortage of medical equipment, such as ventilators, as well as PPE, like face masks, sanitizers, and gloves. This shortage can be attributed to a lack of planning from the healthcare sector in the case of a pandemic [124]. However, many private companies are now supporting the government to expiate this. Even though the private healthcare sector is fully prepared for every eventuality, unlike other sectors, the sector is facing two major challenges:

1. Investing additional manpower, equipment, consumables, and other resources to ensure 100% preparedness for safety in the hospitals and eventual treatment of patients.
2. Experiencing a sharp drop in the Outdoor Patient Department (OPD) patients, elective surgeries, and international patients.

The industry is also likely to benefit from increased awareness about healthcare. There should be increased recruitment of medical professionals, and front-line health workers should be prioritized in receiving vaccines and diagnostic tests, as well as the families of these workers, due to the high risk of transmission. Start-ups that aim to develop health-related products will receive more attention from investors, creating a series of biotechnology ventures that are inspired by the COVID-19 experience.

As people have grown to realize the importance of healthcare, more students are drawn to health-related education. In such a critical time, one of the only industries that have shown no signs of slowing down is the healthcare sector, making proper healthcare the only way to make it to the other side of this pandemic. With this field having the greatest demand in such a situation, many students will be attracted to healthcare fields.

### 3.5. Pharmaceutical Response

To expedite the much-needed process of clinical trials for COVID-19 treatments, as mentioned previously, National Institute of Health (NIH) has collaborated with 16 companies, including AbbVie, Amgen, AstraZeneca, Bristol Myers Squibb, Evotec, GlaxoSmithKline, Johnson & Johnson, KSQ Therapeutics, Eli Lilly, Merck, Novartis, Pfizer, Roche, Sanofi, Takeda, and Vir Biotechnology. While big pharmaceutical companies usually work independently, these industries are now collaborating with different companies and laboratories in the fight against COVID-19. Such NIH industry partnership allows for standardized testing protocols and gives researchers access to better facilities for diagnoses, treatment, and vaccine testing [125]. These industrial partners have promising potential results of their respective research in the fields of diagnostics, prevention, and treatment, as highlighted in Table 1.

## 4. Conclusions

In this paper, the biological background of coronaviruses was provided, with a specific focus on the newly emerging virus SAR-CoV-2 and the ongoing pandemic COVID-19. The disease’s symptoms, methods of transmission, and the currently available medical supportive care were discussed. The control measures for a pandemic were highlighted to incorporate herd immunity, shield immunity, drug repurposing, and vaccine development. The various tools of protection were demonstrated, along with their proper use. A unique perspective was provided to utilize DIY fabrication methods to make face masks, face shields, and ventilators that provide adequate cost-effective protection against the disease. A multidisciplinary approach was presented to integrate machine learning applications, statistical simulations, and digital monitoring in combating COVID-19, tracking its spread, and predicting its trajectory. The role of public regulations in controlling the disease infection rate was shown, with specific emphasis on standardizing the criteria to report new COVID-19 patients, developing novel vaccines, and running nation-wide immunity tests. Finally, this review emphasized the need for preparedness for the management of the COVID-19 pandemic and beyond.

## Figures and Tables

**Table 1:** This table highlights prominent collaborations of pharmaceutical companies and details on what these companies are working on about COVID-19 diagnostics, prevention, and treatment.

Company	Field of COVID-19 Work	Description of Work
Abbott	Diagnostics	Working on molecular (m2000 and ID NOW) and antibody tests [126]
Abbvie	Treatment	Adopting its antiretroviral therapy of HIV (Lopinavir/Ritonavir) for COVID-19 [127]
Amgen and Adaptive Biotechnologies	Diagnostics and Treatment	Researching neutralizing antibodies to prevent and treat COVID-19 [128]
AstraZeneca	Diagnostics, Prevention, and Treatment	Collaborating with different university labs to manufacture COVID-19 diagnostics, treatment, and vaccines [129]
BioNTech and Pfizer	Prevention	Collaborating on a COVID-19 vaccine [130]
Bristol Myers Squibb	Treatment	Collaborating with the community to accelerate therapies via potential molecule screening and testing current medications [131]
Eli Lilly	Treatment	Adopting its rheumatoid arthritis therapy (Baricitinib) and other antibody therapies for COVID-19 [132]
Evotec	Diagnostics and Treatment	Collaborating with the community to accelerate therapies via potential antibody screening [133]
Gilead	Treatment	Adopting its antiviral therapy (Remdesivir) for COVID-19 [134]
GlaxoSmithKline and Sanofi (GSK)	Prevention	Collaborating on a COVID-19 vaccine using GSK’s pandemic adjuvant technology and Sanofi’s S-protein COVID-19 antigen [135]
Johnson & Johnson and Janssen	Prevention	Collaborating on a COVID-19 vaccine using Janssen’s AdVac^®^ and PER.C6^®^ technology [136]
KSQ Therapeutics	Treatment	Using its CRISPRomics^®^ to discover targets for the current COVID-19 treatments [137]
Merck	Prevention and Treatment	Researching the molecular mechanism of COVID-19 to identify potential vaccine and medicine targets for COVID-19 [138]
Moderna	Prevention	Collaborating on a COVID-19 vaccine using mRNA technology [139]
Novartis	Prevention	Adopting its autoimmune disease therapy (Canakinumab) for COVID-19 [140]
Roche	Diagnostics and Treatment	Researching COVID-19 antibody tests [141]
Takeda, CSL Behring, Biotest, BPL, LFB, and Octapharma	Treatment	Adopting hyperimmune globulin for COVID-19 treatment [142]
Vir Biotechnology	Diagnostics and Treatment	Researching neutralizing antibodies to prevent and treat COVID-19 [143]
